# The Brain Activation of Two Motor Imagery Strategies in a Mental Rotation Task

**DOI:** 10.3390/brainsci15010008

**Published:** 2024-12-25

**Authors:** Cancan Wang, Yuxuan Yang, Kewei Sun, Yifei Wang, Xiuchao Wang, Xufeng Liu

**Affiliations:** Department of Military Medical Psychology, Air Force Medical University, Xi’an 710032, China; wangcan@fmmu.edu.cn (C.W.); yangyuxuan@fmmu.edu.cn (Y.Y.); sunkewei@fmmu.edu.cn (K.S.); wangyifei6@fmmu.edu.cn (Y.W.); wangxc123@fmmu.edu.cn (X.W.)

**Keywords:** motor imagery, visual imagery, kinesthetic imagery, mental rotation, fNIRS

## Abstract

**Background:** Motor imagery includes visual imagery and kinesthetic imagery, which are two strategies that exist for mental rotation and are currently widely studied. However, different mental rotation tests can lead to different strategic performances. There are also many research results where two different strategies appear simultaneously under the same task. Previous studies on the comparative brain mechanisms of kinesthetic imagery and visual imagery have not adopted consistent stimulus images or mature mental rotation paradigms, making it difficult to effectively compare these types of imagery. **Methods:** In this study, we utilized functional near-infrared spectroscopy (fNIRS) to investigate the brain activation of sixty-seven young right-handed participants with different strategy preferences during hand lateral judgment tasks (HLJT). **Results:** The results showed that the accuracy of the kinesthetic imagery group was significantly higher than that of the visual imagery group, and the reaction time of the kinesthetic imagery group was significantly shorter than that of the visual imagery group. The areas significantly activated in the kinesthetic imagery group were wider than those in the visual imagery group, including the dorsolateral prefrontal cortex (BA9, 46), premotor cortex (BA6), supplementary motor area (SMA), primary motor cortex (BA4), and parietal cortex (BA7, 40). It is worth noting that the activation levels in the frontal eye fields (BA8), primary somatosensory cortex (BA1, 2, 3), primary motor cortex (BA4), and parietal cortex (BA40) of the kinesthetic imagery group were significantly higher than those in the visual imagery group. **Conclusion:** Therefore, we speculate that kinesthetic imagery has more advantages than visual imagery in the mental rotation of egocentric transformations.

## 1. Introduction

### 1.1. The Connection Between Mental Rotation and Motor Imagery

Mental rotation tasks have become a mature paradigm for studying cognitive processes [1]. There are different strategy classifications in mental rotation. Strategies can be divided into piecemeal strategy and holistic strategy based on visual perspectives [2], or into egocentric strategy and objectcentric strategy based on reference frames [3]. Furthermore, since some stimulus images involve mental representations, mental rotation can also be executed using motor imagery strategies [4,5]. Motor imagery (MI) refers to the intentional execution of a certain action by the brain without actual movement. Its brain activation has been found to be very similar to that during actual movement, and can be divided into visual imagery (VI) and kinesthetic imagery (KI) [6,7]. VI visualizes the movement of objects by participants without the need to perceive muscle activity, while KI involves an individual’s perception of muscle movement [7]. Participants adopt different strategies when they face different types of stimuli. For the object stimulation images of mental rotation (such as letters, numbers, or cubes), they tend to use VI, which completes the rotation of the object from a third-person perspective [8,9]. At the same time, for subject stimulation images involving body parts (such as hands, feet, or the whole body), participants tend to use KI, which imagines in their minds that their body parts are rotating [10,11,12]. We can find that this difference is largely influenced by the properties of the images, and stimuli involving their own body parts are undoubtedly special.

However, the occurrence of strategies is not necessarily solely influenced by the type of stimulus. There is not only a single type of motor imagery strategy for both object and subject stimuli in mental rotation, as it is also influenced by the participant’s own choice of strategy type [13]. On the one hand, Vingerhoets et al. found that in addition to body parts, rotating operable objects (such as tools) can also activate the movement area [14]. That is to say, participants will imagine themselves using their hands to manipulate objects, achieving the same effect as the brain activation of their hands, which proves that the they also use KI for object stimuli. Ankaoua and Luria also found that participants exhibited very similar behaviors and ERP time processes when using chairs and hands as stimuli [15]. On the other hand, after reviewing the previous literature, ter Horst et al. found that young people use VI in some studies when seeing pictures of the back of their hands [16]. In this case, hand stimulation is only visually rotated. Nagashima et al. also confirmed this point, as they found that some participants chose VI when facing images of the back of their hands [17,18]. Mibu et al. conducted a more detailed analysis at the individual level and found that up to 50% of participants used KI when faced with palm images, and 22% of participants chose KI when using images of the backs of hands as stimuli [19]. This suggests that the proportion of participants using KI for their palms was not as high as expected. In addition, a meta-analysis of functional neuroimaging studies also showed that MR activated regions in the posterior cingulate and motor prefrontal cortex, which are similar to those involved in movement, with strong neural-substrate overlap [20]. However, not all tasks included in the analysis activate the motor cortex, which suggests that different strategies are adopted by participants in response to the same or different stimuli. Therefore, whether or not the motor area is activated might depend on the participant’s strategy preference, rather than being entirely determined by the stimulus itself.

### 1.2. Neural Activity of VI and KI Strategies

In motor imagery, VI and KI are different cognitive processes, although there is some overlap in the activated brain regions, each relying on different brain circuits with distinct functional roles [1,6,7,21]. Dijkstra et al. reviewed previous research on VI and found that the activation area of VI includes the visual, parietal, and prefrontal cortices, and to some extent depends on visual simulation processes, so it is similar to the brain activation during visual perception [22]. However, while certain brain regions involved in high-level visual perception are activated, the extent of visual-related activity depends on the stimulus and the demands of the task [23]. Amemiya et al.’s study found that both healthy participants and blind participants had their bilateral prefrontal cortices, including the supplementary motor area (SMA), activated when they walked or jogged within a 2 m radius using KI [24]. After the transcranial magnetic stimulation (TMS) of the primary motor cortex (M1 area) by Pelgrims et al., the ability to distinguish between left and right in the hand-rotation picture test was significantly impaired and reaction time was significantly increased, indicating that the M1 area is an important neural circuit for motor imagery [25]. Thus, VI primarily activates the parietal lobe and visual cortex, while MI involves the greater activation of the motor area [26].

However, these findings of brain activation triggered by the two types of strategies are not always the case. VI was found to elicit activation in premotor and supplementary motor areas during the mental rotation of letters and arms [6]. Hyung Lee et al. asked participants to imagine grasping and letting go of a target and also found that VI, like KI, involved the significant activation of the left premotor area and inferior parietal lobe [27]. In addition, the dorsolateral prefrontal lobe is part of the cognitive and motor network and plays an important role [28]. In this case, frontal brain regions were found to differ significantly between the KI and VI groups. In particular, the analysis of evoked responses showed that the activity of the frontal cortex was suppressed during MI in all participants who used KI, whereas in participants who used VI the frontal cortex was always active [7]. However, the latest study from Kwon et al. showed that VI causes stronger activation in the right frontal lobe, while KI causes greater activation in the left frontal lobe [29]. In conclusion, the discussion of KI and VI continues and remains inconsistent.

To summarize, many of the comparative studies on KI vs. VI have not used consistent stimulus images or well-established mental rotation paradigms to compare the two strategies, so it is difficult to make valid comparisons between different results. That is, there is a lack of comparative studies of the two strategies under the same mental rotation test. Therefore, our study used the hand laterality judgment task (HLJT) as the stimulus material because it is a commonly used stimulus material for mental rotation and a common task for measuring motor imagery ability [17,19,21,30]. We hypothesized that the presence of the two sets of strategies would be equivalent under these conditions. Then, we utilized near-infrared spectroscopy (fNIRS) techniques with high ecological validity to probe brain activation between different motor imagery strategies and differences between KI and VI under the same stimuli.

## 2. Materials and Methods

### 2.1. Participants

We recruited seventy healthy, right-handed male college students to participate in the experiment, excluding physical conditions such as color-vision deficits and limb injuries or mobility limitations. Before the fNIRS recording began, we had participants practice the task for a period of time until they felt familiar with it. After that, they were asked to give a verbal report of their strategies. At this point, participants were asked: “What method do you use to judge whether the image is left or right hand?”. After participants reported their strategies, they were divided into two groups for the fNIRS recording according to their strategy preferences. The self-report of participants using VI was usually as follows: “I would determine whether the picture was left or right hand based on the orientation or position of the thumb. If it’s the palm of the hand, thumb on the left is the left hand, and thumb on the right is the right hand. If it’s the back of the hand, it’s the opposite”. The self-report of participants using the KI was usually the following: “I would visualize the picture as being my own hand and then use one of my own hands to compare it to the picture”. They were required to continue using the reporting strategy to complete the next task. And at the end of the fNIRS recording, we had participants give a verbal report of their strategy again to ensure that they had performed the task in accordance with the realization of the agreed-upon strategy. After excluding participants with poor blood oxygenation signals, the final number of participants included in the data analysis was sixty-seven, with thirty-four participants (age: 19.32 ± 1.56) in the VI group and thirty-three participants (age: 19.47 ± 1.21) in the KI group. All participants volunteered, signed an informed consent form, and received some payment at the end of the experiment.

### 2.2. Procedure

**Figure 1:**
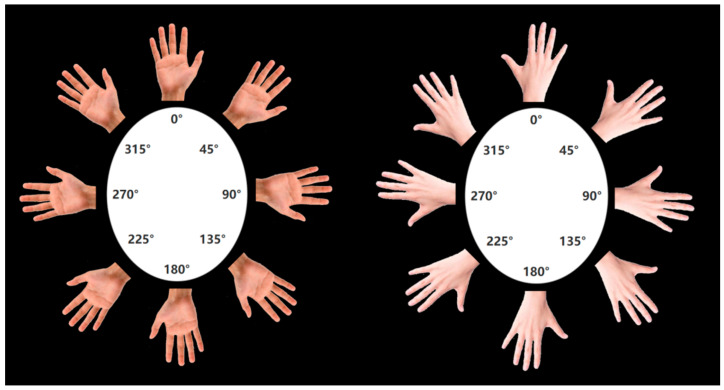
Stimulus materials (right hand as an example).

Each participant performed the hand laterality judgment task (HLJT) while sitting in front of a computer screen in a quiet room with normal lighting conditions. They were at a uniform distance from the center of the screen and had their left index finger placed on the Z button on the keyboard and their right index finger placed on the M button on the keyboard. E-Prime 3.0 (Psychology Software Tools, Inc., Pittsburgh, PA, USA) was used to present the stimulus pictures. The stimulus pictures were of the hand in its natural state and consisted of 32 sheets: 2 (left hand/right hand) × 2 (palm hand/back of hand) × 8 (rotation angles: 0°, 45°, 90°, 135°, 180°, 225°, 270°, 315°) (see Figure 1). Participants were required to respond as quickly and accurately as possible to whether the stimulus was a left or right hand by pressing one of the two defined keys on the computer keyboard with their index finger after a picture was presented. If the stimulus picture was a left hand, they needed to press the “z” key, and if the stimulus picture was a right hand, they needed to press the “m” key. Participants were not allowed to look at or move their hands during the entire procedure.

**Figure 2:**
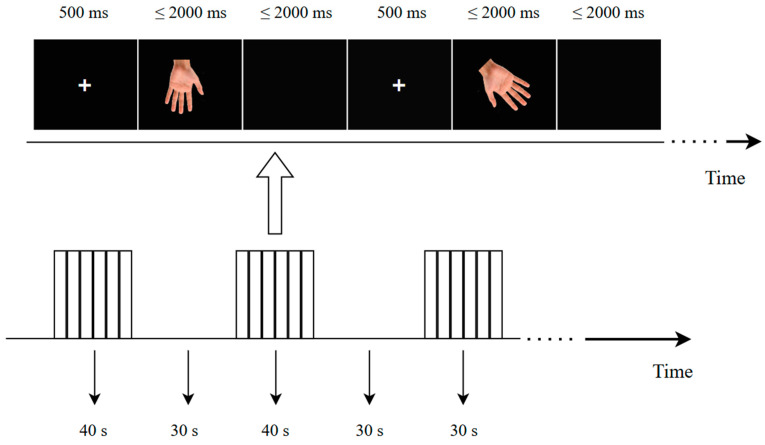
Experimental procedure (“+”: central fixation across).

The fNIRS recording used a block design for the HLJT, where each block was a 40 s fixed question–answer period followed by a 30 s resting break. Question answering and rest alternated 8 times (see Figure 2). Each block contained 16 trials. At the beginning of each trial, a 500 ms central fixation cross was randomly presented in the center of the screen, followed by the presentation of the stimulus picture. Participants were required to make a quick and accurate keypress response after seeing the stimulus. Key presses were followed by a stimulus interval; if the participant had not pressed a key for more than 2000 ms, the stimulus disappeared and the participant was moved on to the next trial. The length of the stimulus interval depended on the duration of the participant’s response, and the two durations’ combined length was fixed at 2000 ms.

The experiment was a one-way two-level (groups: VI group, KI group) between-subjects design. In the behavioral data analysis, the dependent variables were the mean reaction time for correct responses and the percentage of correct responses. In the fNIRS data analysis, the dependent variable was the relative amount of change in the concentration of oxyhemoglobin (HbO) in the participants, because HbO is more sensitive to changes in task stimuli [31,32,33]. The sampling rate of the instrument in this study was 41.67 Hz.

**Figure 3:**
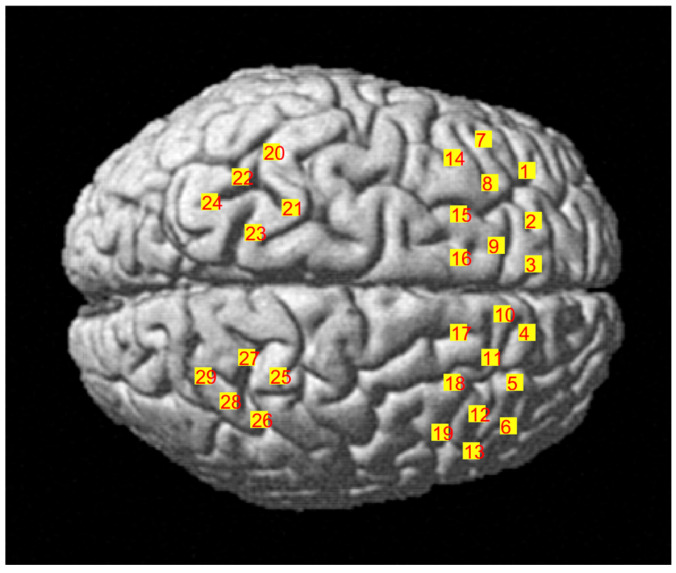
Channel layout of fNIRS.

Changes in subregional oxyhemoglobin concentration (HbO), deoxyhemoglobin concentration (HbR), and total hemoglobin concentration (HbT) were detected using the LABNIRS functional near-infrared brain imager (Shimadzu Corporation, Kyoto, Japan). We used 29 channels consisting of 12 sources (emitting 3 wavelengths of near-infrared light at 780, 805, and 830 nm) and 12 detectors in a 2 × 7 optode probe set and a two 2 × 3 optode probe set, with a probe spacing of 3 cm (see Figure 3). Referring to the probe layout method of previous studies, covering the main activated brain areas of the task (frontal, motor, and parietal lobes), the probes were placed according to the international 10-10 EEG system [34,35]. Cz, Nz, AL, AR points and probe positions were determined using a 3D localizer (FASTRAK, Polhemus, Colchester, VT, USA). The fNIRS channel positions were aligned to the MNI spatial coordinates by a probabilistic alignment method to obtain correspondence with the Brodmann area (see Table 1).

### 2.3. Data Analysis

Independent samples *t*-tests were performed on the behavioral data of the two groups of participants using SPSS 26.0 (SPSS Inc., Chicago, IL, USA). We defined reaction time as the time between the beginning of the stimulus and the key press, focusing only on the correct rate of each participant and the reaction time of the correct response, the same as in previous HLJT studies [18,19,36]. If the reaction time exceeded 2 s, it was considered an incorrect reaction.

The processing of hemoglobin change signals from functional near-infrared brain imaging used NIRS_SPM software running on Matlab (R2022b, The MathWorks Inc., MA, USA) [37]. NIRS_SPM is a plug-in for Matlab which mainly analyzes fNIRS data based on the general linear model (GLM). During the preprocessing of the data, noise (head movement, heartbeat, respiration, etc.) and drift were excluded according to the Hemodynamic Response Functions (HRF) and Wavelet-MDL methods [38]. Relevant studies have used both methods and the denoising effect has been better confirmed [39]. In addition, a precoloring method was chosen for this study where the kernel function could be either a Gaussian filter or an HRF (Hemodynamic Response Function) low-pass filter [40]. The band-pass filter was set to 0.01–0.1 Hz. After preprocessing, data from three participants were excluded due to excessive artifacts. Then, the general linear model (GLM) was used to integrate task effect, and a task-fitting reference wave was used to infer parameter estimation (weight of beta value in the GLM model). After parameter estimation, beta values were obtained for each channel of each participant. We calculated the total mean of each channel and performed the pooling of the two groups, and then performed a one-sample *t*-test and an independent-sample *t*-test for the beta values obtained under different motor imagery strategies. Finally, all *p*-values were corrected using FDR (false discovery rate), with *p* < 0.05 being significant [41].

## 3. Results

### 3.1. Behavioral Results

An independent samples *t*-test was performed on the mean response times and correctness of responses for both groups of participants (see Table 2). We found that the mean reaction time for correct responses in the task was significantly longer for participants in the VI group than for participants in the KI group (*p* < 0.01). Although the accuracy of participants in the KI group was higher than the accuracy of participants in the VI group, the difference was not significant (*p* < 0.11).

### 3.2. fNIRS Results

**Figure 4:**
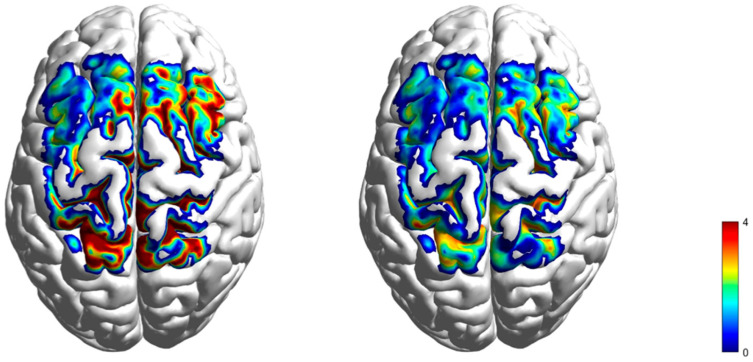
Rendering of *t*-value of cortical activation in two groups ((**left panel**) KI, (**right panel**) VI).

A one-sample *t*-test with a test value of 0 was performed on the beta values averaged across channels during the task state for participants in the KI and VI groups, and then brain renderings were plotted based on the t-values obtained by the two groups in each channel (see Figure 4). After FDR correction, 24 channels were significantly activated in the KI group of participants in the task state, 17 channels were significantly activated in the VI group of participants in the task state, and there were 15 overlapping activated channels (*p* < 0.05, see Table 3). The activated regions in both groups of participants contained the dorsolateral prefrontal cortex (BA9, 46), premotor cortex (BA6), supplementary motor area (SMA), primary motor cortex (BA4), and parietal cortex (BA7, 40). More brain regions were activated in the KI group than in the VI group. After comparing the channels that were jointly significantly activated by the two groups, it was found that the *t*-values of the KI group were all higher than those of the VI group.

**Figure 5:**
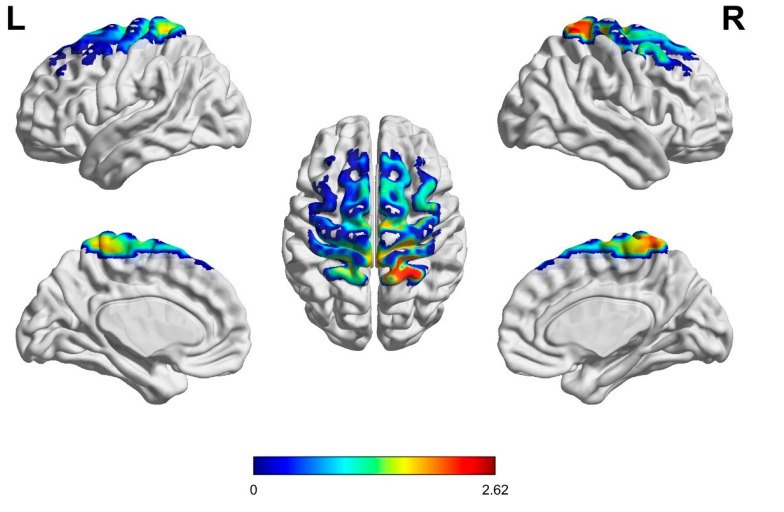
Comparison of *t*-values of cortical activation in two groups (L: Left brain; R: Right brain).

**Figure 6:**
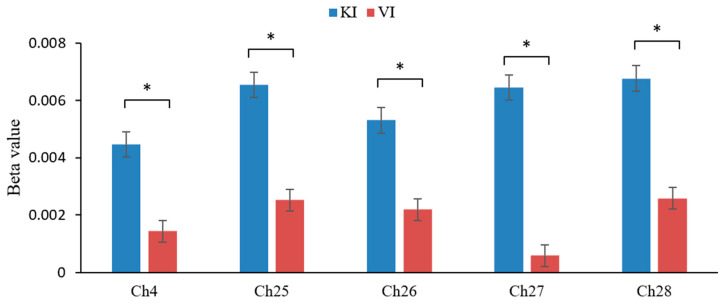
Beta values of cortical channels with significant activation differences between two groups (* *p* < 0.05).

Independent samples *t*-tests were performed on the beta values of each channel during the task for participants in group KI and participants in group VI on a channel-by-channel basis, and renderings were plotted based on the *t*-values obtained (see Figure 5) After FDR correction, the channels with significant activation differences between the two groups were Ch4, Ch25, Ch26, Ch27, Ch28 (see Figure 6). This indicates that the KI group showed significantly higher activation in the frontal eye fields (BA8), primary somatosensory cortex (BA1, 2, 3), primary motor cortex (BA4), and parietal lobe (BA40) than the VI group.

## 4. Discussion

The neural activation involved in KI and VI is an interesting topic in the field of psychological research. The aim of this study was to investigate the behavioral performance and neural mechanisms generated by different motor imagery strategies in mental rotation by using brain imaging techniques, focusing on the brain regions activated by KI and VI and their differences. For this purpose, we chose the hand laterality judgment task that has been widely used in both mental rotation and motor imagery studies [5,11,30,42,43].

Before the formal experiment, we fully informed the participants about the experiment and confirmed their strategy preferences, and after the conclusion of the experiment we again conducted a plausibility check that the strategies were performed as required. Previous studies have also controlled for strategy, but they have controlled participants to use the specified strategy through guidelines and did not ask participants afterward whether they performed the task as required during the procedure [26,27,29,44]. In addition, we prescribed the corresponding strategies to the participants based on their strategy preferences, avoiding the added burden of using a motor imagery strategy that the participants did not specialize in. The participants’ self-reported results also corroborated our hypothesis that the two motor imagery strategies coexisted under the mental rotation of the participant’s stimulus. Notably, we found in participants’ self-reports the importance of having an external referent in VI, such as a thumb. Researchers have also added a dot as a marker on one of the fingertips in the stimulus image, guiding participants to use the middle finger as a referent and distinguish whether the image is a left hand or a right hand by judging the direction of the dot relative to the middle finger. This demonstrates that an external referent is quite important in VI [44]. However, in KI, there is no need for an external referent because the participant feels the stimulus with themselves.

### 4.1. Discussion of Behavioral Results

Our behavioral findings revealed that the mean reaction time for correct responses in the HLJT was significantly longer for participants in the VI group than for participants in the KI group; although there was also a difference in correctness rates between the two groups, it was not significant. This suggests that the participants who used KI in correct responses responded faster than those who used VI. In addition, KI does not affect the correct rate because there is no significant difference between the correct rate of the KI and VI groups, even though the correct rate of the KI group is higher than that of the VI group. Therefore, we hypothesized that this might indicate a potential advantage of KI in the mental rotation of subject stimuli. This can be demonstrated in some comparative studies between athletes and non-athletes. The results of many studies have shown that some athletes have an advantage in egocentric transitions, that is, in contexts where KI is used more often [45,46,47]. This may be because athletes are inherently required to perceive, encode, and translate spatial information more acutely and accurately in sport compared to the general population, and are required to accomplish a number of rotations and activities involving the body. They are more likely to automatically connect their nervous system to sensory information during mental rotation tasks, helping to demonstrate strengths consistent with KI characteristics [48]. The mechanisms for this dominance are the neural correlates of motor and mental training [49].

### 4.2. Discussion of fNIRS Results

The parietal lobe is unanimously recognized by researchers as the central region of mental rotation [32,50,51]. In our channel layout, the parietal lobe region included the supramarginal gyrus part of Wernicke’s area (BA40) and the somatosensory association cortex (BA7). When the participants in the KI group underwent mental rotation of the main stimulus, significant activation occurred in the dorsolateral prefrontal cortex (BA9, 46), premotor cortex (BA6), supplementary motor area (SMA), primary motor cortex (BA4), and parietal cortex (BA7, 40) of the brain. This result can be supported by previous studies. For example, Ehrsson et al. asked healthy participants to explicitly imagine their hands in motion, and the results showed that some motor areas of the participants were significantly activated, including the primary motor cortex, premotor cortex, and supplementary motor area [52]. In addition to the above regions, the dorsolateral prefrontal cortex and parietal lobe have also been found to be part of cognitive and motor networks [28]. This suggests that the participants were successful in mental rotation using KI and that the relevant motor regions were involved in perception, planning, and control. Meanwhile, VI was found to activate the same brain regions as KI, also activating the dorsolateral prefrontal cortex (BA9, 46), premotor cortex (BA6), supplementary motor area (SMA), primary motor cortex (BA4), and parietal cortex (BA7, 40), but the activated regions were smaller than in KI. As has been noted, the network of areas activated in KI and VI during a mental rotation task of the arm shows substantial but incomplete overlap [6]. A meta-analysis with an activation likelihood estimation algorithm also reported that KI and VI activate the same regions [53]. Therefore, it can be hypothesized from our results that VI and KI share similar neural networks, except that KI triggers brain activation in a wider range of brain regions with a higher degree of activation.

After comparing brain activation between the KI and VI groups, it was found that the KI group had significantly higher activation in the frontal eye fields (BA8), primary somatosensory cortex (BA1, 2, and 3), primary motor cortex (BA4), and parietal lobe (BA40) than the VI group. As mentioned in previous studies, KI consumes more brain resources than VI [54]. Nobuaki et al. have shown that the vividness of MI is related to the activity of the right orbitofrontal cortex [55], which may help us to explain the performance of the KI group in the frontal eye fields (BA8). And we can also hypothesize that the vividness of MI relies mostly on the contribution of KI. The activation of the primary somatosensory cortex could indicate that the subject stimulus induces a somatosensory sensation in participants who perceive the need for motor planning, and that the generation of this planning affects somatosensory areas and is more pronounced in KI. In addition, it has been found that faster mental rotation is associated with sensorimotor activity [56]; our behavioral finding that the KI was faster than the VI group is fully explained here. And the KI group showed significantly higher activation in the primary motor cortex than the VI group, again revealing the important role of the primary motor cortex associated with motor execution in KI [13]. As one study found, a condition of hand muscle twitching increased response times to a task of lateralized judgment, but did not affect a task of equal difficulty (e.g., letters) [25]. This indicates a critical role for the primary motor cortex in MI and especially in KI. In addition, the stronger activation of the parietal lobe in KI indicates on the one hand that this strategy requires a greater expenditure of resources in mental rotation, and on the other hand reflects the fact that KI requires more attention than VI. Thus, the neural mechanisms of these two strategies still differ.

## 5. Limitations

Considering the following limitations of this study, the interpretation of the results should be made with caution. First, based on previous research, gender differences have always been a commonly discussed as an influencing factor in mental rotation, and there is still controversy [4,57,58]. Therefore, we attempted to avoid the influence of gender factors in this experiment. Our study only explored the performance of male participants, so the phenomena observed in the results can only be used to explain male participants. We also need to recruit females in future research and consider the influence of factors such as hormone levels and the menstrual cycle [59,60]. Second, multi-channel fNIRS has lower spatial resolution compared with fMRI, although the fNIRS channel set according to the international 10–20 system can confirm the difference between Brodmann’s areas. And the above analyses are based on the group level, including the channel localization obtained from the optode placement, so the optode variations among different participants may lead to biases in the activation area, which could affect the results. Moreover, it is worth noting that due to the limited number of optical poles in the fNIRS, we did not focus on activation in occipital lobe regions that have less controversy. In reviewing the previous literature, we found that the activation of occipital lobe regions attributed to VI is responsible for processing visual information [13,26,29]. VI largely involves the network between the frontal and parietal lobes and the visual cortex, and the excitability of the primary visual cortex is highly dependent on the participation of VI and is related to its accuracy [55,61]. Due to our limited exploration of visual cortex activation for VI, the completeness of our neural analysis may be diminished.

## 6. Conclusions

We investigated brain activation differences between two groups of participants employing different motor imagery strategies under a uniform mental rotation subject stimulus target in a young population. Prior to the formal study, we examined the participants’ strategy preferences and learned about the specific imagery methods used by the participants in the two strategy groups. In the formal experiment, we set the frontal and parietal lobes as well as the motor-related regions as the brain regions of interest, and in the results we found that both KI and VI significantly activated the dorsolateral prefrontal cortex (BA9, 46), premotor cortex (BA6), supplementary motor area (SMA), primary motor cortex (BA4), and parietal cortex (BA7, 40). However, the activation area and degree of KI was greater than that of VI because KI triggered significantly more brain activity in the frontal eye fields (BA8), primary somatosensory cortex (BA1, 2, 3), primary motor cortex (BA4), and parietal lobe (BA40) than VI. This suggests that, although the two may have similar brain activation networks, their neuropsychological mechanisms are not the same. Moreover, the faster performance of KI than VI that we found in terms of reaction time may reflect the dominance of KI activation in the primary somatosensory cortex.

## Figures and Tables

**Table 1:** Correspondence between fNIRS channel and Brodmann area.

Brodmann Area	Channel
Dorsolateral prefrontal cortex (BA9, 46)	Ch1, 2, 5, 6, 8, 12, 13, 14
Includes frontal eye fields (BA8)	Ch3, 4, 9, 10, 11, 15, 18
Pars opercularis part of Broca’s area (BA44)	Ch7
Pre-motor and supplementary motor cortex (SMA) (BA6)	Ch16, 17, 19
Primary somatosensory cortex (BA1, 2, 3)	Ch20, 23, 26, 27
Primary motor cortex (BA4)	Ch21, 25
Supramarginal gyrus part of Wernicke’s area (BA40)	Ch22, 28
Somatosensory association cortex (BA7)	Ch24, 29

Note: The channel locator areas listed in this table are the areas with the highest probability of coverage for that channel.

**Table 2:** The differences in performance between the two groups of participants (M ± SD).

	VI Group	KI Group	*t*	*p*
*N*	34	33		
Reaction time (ms)	917 ± 179	794 ± 126	3.25	<0.01
Accuracy (%)	87.85 ± 9.76	91.18 ± 6.52	−1.64	0.11

**Table 3:** Comparison of brain activation in KI and VI groups (HbO).

Brodmann Area	Channel	MNI (x y z)	Percentage of Overlap	*t* (KI Group)	*t* (VI Group)	*p* (Uncorrected in KI Group)	*p* (FDR-Corrected in KI Group)	*p* (Uncorrected in VI Group)	*p* (FDR-Corrected in VI Group)
Dorsolateral prefrontal cortex	1	−38 42 38	56%	1.66	1.68	0.11	0.12	0.11	0.13
	2	−21 43 50	63%	2.44 *	1.61	0.02	0.03	0.12	0.14
	5	30 38 51	56%	3.17 *	2.05	<0.01	0.01	0.05	0.07
	6	45 36 41	52%	3.72 *	2.11	<0.01	<0.01	0.05	0.06
	8	−33 30 53	68%	1.09	1.69	0.29	0.29	0.11	0.13
	12	40 26 54	74%	4.55 *	3.21 *	<0.01	<0.01	<0.01	0.02
	13	51 23 43	53%	4.98 *	2.94 *	<0.01	<0.01	0.01	0.02
	14	−43 18 56	65%	1.83	2.41 *	0.08	<0.01	0.03	0.04
Primary somatosensory cortex	20	−43 −39 66	75%	4.60 *	3.61 *	<0.01	<0.01	<0.01	0.02
	23	−18 −46 78	51%	5.35 *	2.65 *			0.01	0.03
	26	42 −45 67	74%	4.39 *	3.33 *			<0.01	0.02
	27	21 −48 76	51%	4.39 *	0.29			0.77	0.77
Primary motor cortex	21	−26 −35 75	58%	3.96 *	1.64	<0.01	<0.01	0.12	0.14
	25	28 −39 75	60%	4.89 *	3.21 *			<0.01	0.02
Includes frontal eye fields	3	−7 44 55	82%	2.10	2.46 *	0.05	0.05	0.02	0.04
	4	14 42 56	83%	4.38 *	2.17	<0.01	<0.01	0.04	0.06
	9	−15 33 60	100%	2.10	2.22	0.05	0.05	0.04	0.06
	10	7 34 61	91%	3.94 *	3.18 *	<0.01	<0.01	<0.01	0.02
	11	22 30 60	100%	3.89 *	2.75 *	<0.01	<0.01	0.01	0.03
	15	−25 20 65	80%	2.74 *	1.57	0.01	0.02	0.13	0.14
	18	31 17 64	69%	3.83 *	3.20 *	<0.01	<0.01	<0.01	0.02
Pre-motor and supplementary motor cortex	16	−9 21 68	61%	5.25 *	2.22	<0.01	<0.01	0.04	0.06
	17	15 20 68	71%	3.93 *	3.30 *			<0.01	0.02
	19	46 14 56	46%	6.69 *	3.64 *			<0.01	0.02
Pars opercularis part of Broca’s area	7	−49 28 39	81%	2.62 *	2.51 *	0.02	0.02	0.02	0.04
Somatosensory association cortex	24	−27 −60 70	87%	4.62 *	3.07 *	<0.01	<0.01	0.01	0.02
	29	28 −61 71	95%	4.20 *	1.22			0.24	0.25
Supramarginal gyrus part of Wernicke’s area	22	−36 −50 70	35%	4.33 *	2.78 *	<0.01	0.01	<0.01	0.03
	28	35 −54 69	38%	4.70 *	2.82 *		0.01		0.03

* *p* (FDR-corrected) < 0.05.

## Data Availability

The datasets presented in this article are not readily available because the data are part of an ongoing study. Requests to access the datasets should be directed to the first author’s email addresses.
